# The congenital popliteal vasculature patterns in fibular free flap reconstruction by means of surgical anatomy in cadavers

**DOI:** 10.1038/s41598-021-99203-1

**Published:** 2021-10-01

**Authors:** Mathee Ongsiriporn, Piyawadee Jongpradubgiat, Sasiprapa Pisittrakoonporn, Natthapong Kongkunnavat, Kosin Panyaatisin, Nutcha Yodrabum

**Affiliations:** 1 Department of Anatomy, Faculty of Medicine Siriraj Hospital, Mahidol University, Bangkok, Thailand; 2 Division of Plastic Surgery, Department of Surgery, Faculty of Medicine Siriraj Hospital, Mahidol University, Bangkok, Thailand; 3 Graduate School of Language and Communication, National Institute of Development Administration, Bangkok, Thailand

**Keywords:** Musculoskeletal system, Anatomy, Cardiology, Risk factors

## Abstract

Fibular free flap (FFF) is frequently used for reconstruction requiring vascularized bone. Thus, understanding its vasculature variation is crucial. This study investigates the popliteal artery branching variations in Thai cadavers and compares them with previous studies. One hundred and sixty-two legs from 81 formalin-embalmed cadavers were dissected. The popliteal artery branching patterns were classified. The previous data retrieved from cadaveric and angiographic studies were also collected and compared with the current study. The most common pattern is type I-A (90.7%). For the variants, type III-A was the majority among variants (6.2%). Type IV-A, hypoplastic peroneal artery, was found in one limb. A symmetrical branching pattern was found in 74 cadavers. Compared with cadaveric studies, type III-B and III-C are significantly common in angiographic studies (p = 0.015 and p = 0.009, respectively). Type I-A is most common according to previous studies. Apart from this, the prevalence of type III-A variant was higher than in previous studies. Furthermore, type III-B and III-C are more frequent in angiographic studies which might be from atherosclerosis. Thus, if the pre-operative CTA policy is not mandatory, the patients at risk for atherosclerosis and population with high variants prevalence should undergo pre-operative CTA with cost-effectiveness consideration.

## Introduction

The fibular free flap (FFF) was introduced in 1975 by Taylor and colleagues^1,2^. This flap was frequently used for reconstructions, especially in defects which require vascularized bone^3^. The fibula is mainly supplied by the peroneal artery (PR) which has a parallel course along the fibula. PR provides the nutrient branch and several periosteal branches^2^. This vascular anatomy allows multiple osteotomy sites in this bone, which is necessary for reconstructive surgery such as mandibular reconstruction^4,5^.

Popliteal artery (PA) and its collaterals, including peroneal artery (PR), anterior tibial artery (AT), and posterior tibial artery (PT), are the main blood supply of the lower leg^5^. The AT arises from PA followed by the tibioperoneal trunk that later divides into PT and PR^6^. In 1989, Kim et al.^6^ proposed a classification modified from Lippert et al. Abou-Foul et al.^5^ proposed additional variants from Kim et al.’s classification. The later classification of Kim et al. together with Abou-Foul et al. are widely used in literature and studies in PA and its collaterals.

Regarding the variation of popliteal artery branching pattern, it has been proposed that embryogenic regression of sciatic artery, a primitive artery of the lower limb bud, and formation of the femoral artery are the antecedent determinants of PA branching variants^7^.

Usually, PR terminates above the ankle joint and contributes less to pedal circulation than AT and PT^8^. However, congenital abnormalities of AT and PT make PR become the dominant blood supply of pedal perfusion^6^. Type III variants, according to Kim et al. and Abou-Foul et al. studies, they categorized as having hypoplastic-aplastic of AT, PT, and type IV, hypoplastic-aplastic PR which precludes harvesting of vascularized FFF^3,9,10^. Harvesting of FFF together with PR in these vascular variants may result in foot ischemia of the donor’s leg or unusable fibular flap, respectively^11^.

Accumulating knowledge regarding PA branching patterns may facilitate surgeons in harvesting vascularized FFF and prevent severe donor site morbidities. To our best knowledge, there are several reports on the differences in prevalence of the popliteal artery variation. These differences do not solely result from ethnicity factor. Furthermore, the reports on PA branching pattern variation in the Asian population are limited^11–13^. In general, the Computed Tomography Angiography (CTA) is a mandatory policy for pre-operative planning of harvesting FFF in several centers. However, in some situations, such as excessive contrast media-level from pre-operative tumor imaging, limited contrast media amount in renal insufficiency patient which requires a separate session of CTA. Therefore, the pre-operative CTA might not be applicable. The cost-effectiveness of pre-operative leg CTA should also be considered^8,14^.

Hence, this study aims to investigate the congenital variation of PA branching by means of cadaveric dissection and compare the prevalence differences between angiographic and cadaveric studies in order to find out the cause of the difference. This will serve as a guidance for the currently controversial decision in pre-operative leg CTA and as baseline information for surgical procedure application**.**

## Results

**Figure 1:**
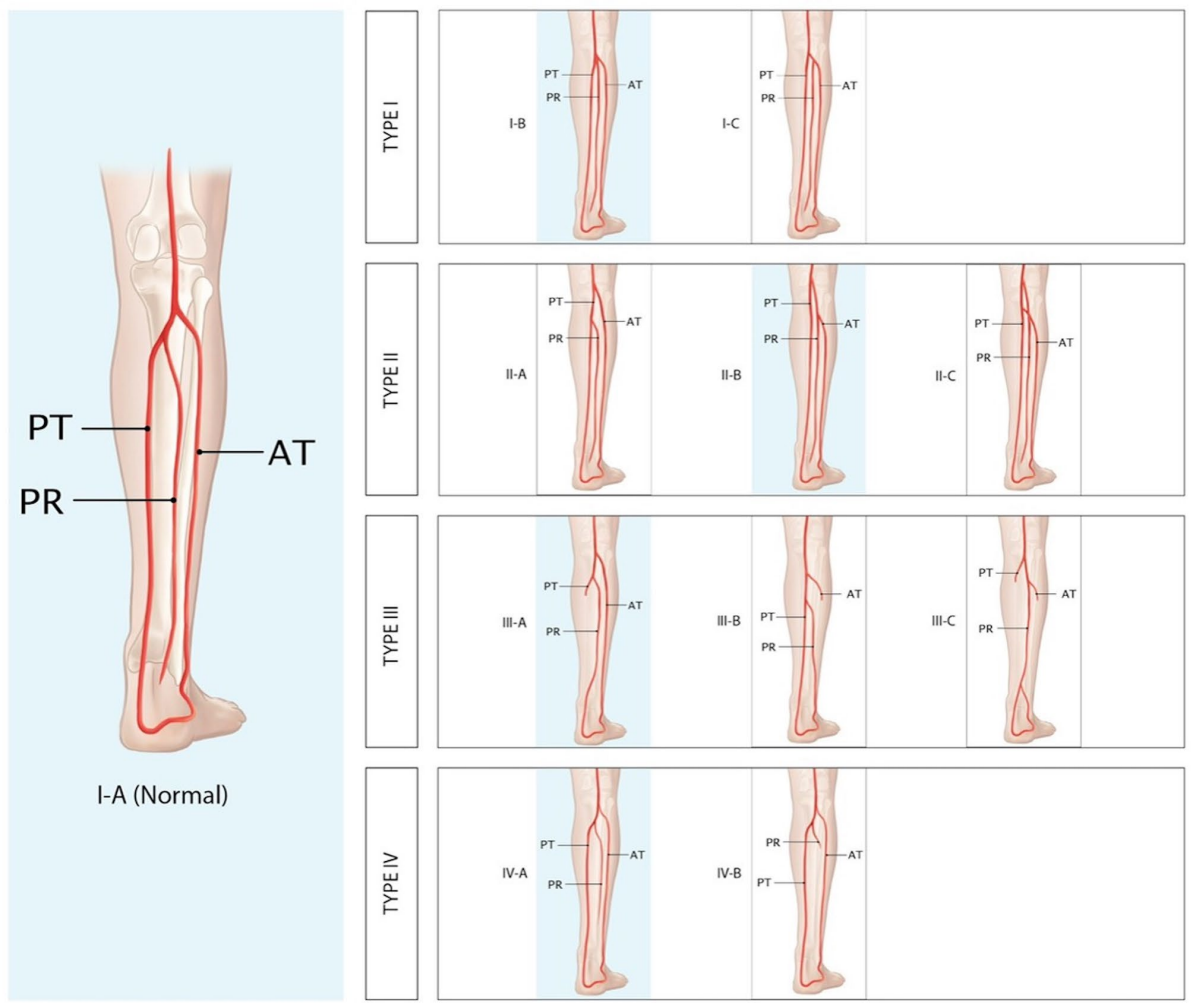
An illustration of popliteal artery (PA) and its branches following classification by Kim et al. and Abou-Foul et al. The popliteal artery branching patterns are classified into 4 major types (I–IV) and divided into 12 subtypes. To note, Type I-A, I-B, II-B, III-A, and IV-A are found in this study (indicated by highlighted in blue rectangles). Type I-A: The common pattern of the popliteal artery and its branches. Type I-B: Trifurcation, AT, PT, and PR arise from the same point, Type I-C: The PT is the first branch, then AT and PR arise from common trunk. Type II-A: The AT arises above the knee joint. Type II-B the PT arises above the knee joint. Type II-C the PR arises above the knee joint. Type III-A: Hypoplastic or aplastic of PT and distal PT replaced by PR. Type III-B hypoplastic or aplastic of AT and dorsalis pedis artery is replaced by PR. Type III-C: hypoplastic or aplastic of both AT and PT results in dominant PR, also called “Peronea arteria magna (PAM)”, which distal PT and dorsalis pedis artery are replaced by PR. Type IV-A: Hypoplastic of PR. Type IV-B: Aplastic of PR. Type III-A and III-B have surgical significance due to the fact that the distal limb will be only supplied by remaining AT or PT, respectively, after FFF harvesting. Type III-C, the dominance of PR without AT and PT supply distal limb, which is contraindicated for FFF harvesting due to the unusable flap. *PA* popliteal artery, *AT* anterior tibial artery, *PT* posterior tibial artery, and *PR* peroneal artery.

One hundred and sixty-two lower extremities were harvested from 81 cadavers. The average age at death is 79.3 years, ranging between 23 and 100 years (Table 1). According to the type of popliteal artery branching, we found that type I-A, the common pattern, was the most common type in this study (90.7%). The variant patterns were found in 15 of 162 limbs (9.3%). Among these variants, the most common variant in this study was type III-A, hypoplasia or aplasia of PT (6.2%), followed by type I-B (trifurcation of AT, PR, and PT), type II-B (a high division of PA), and IV-A (hypoplasia of PR) with the percentage of 1.2%, 1.2%, and 0.6% respectively (Table 2). Other branching patterns, which were not mentioned above, were not found in this study (Fig. 1). We have noticed that type III-A (absent or hypoplastic PT) limbs have a pedal arterial network originating from AT and distal PR, replacing the absence of PT.

**Table 1:** Demographic data of cadavers.

Female N (%)	Male N (%)	Age^a^ years (range)	Weight (kg)	Height (cm)
39 (48.1)	42 (51.9)	79.3 (23–100)	55.3 (33.5–81.5)	157.0 (140–184)

^a^Age at death.

**Table 2:** Variation of popliteal artery branching in lower extremities found in this study.

Branching pattern^a^ (n = 162)	Left leg n(%)	Right leg n(%)	Both legs n(%)
I-A The PA divides into the AT, followed by TPT that later bifurcates into PT and PR	73 (91.1)	74 (91.4)	147 (90.7)
I-B The PA trifurcates into AT, PT and PR	2 (2.5)	0	2 (1.2)
II-B The PA divides into the PT at/above the knee joint, followed by anterior TPT that bifurcates into AT and PR	1 (1.2)	1 (1.2)	2 (1.2)
III-A Hypoplastic-aplastic PT, distal PT replaced by PR	4 (4.9)	6 (7.4)	10 (6.2)
IV-A The PA divides into the AT, followed by TPT that bifurcates into PT and PR. The PR is hypoplasia	1 (1.2)	0	1 (0.6)

Branching pattern^b^ (N = 81)		N (%)	
Symmetrical pattern		74 (91.4)	
Asymmetrical pattern		7 (8.6)	

*PA* popliteal artery, *AT* anterior tibial artery, *PT* posterior tibial artery, *PR*  peroneal artery, *TPT* tibioperoneal trunk.

^a^The classification follows Kim et al. and Abou-Foul et al. Other types which are not shown in the table are not found in this study.

^b^Comparing with contralateral leg, symmetrical pattern of both common pattern and symmetrical variants are included. In terms of the asymmetrical pattern, another limb reveals common pattern in all cases.

When comparing both legs, 74 cadavers had symmetrical arterial branching pattern. Seventy cadavers in the symmetrical pattern group had the type I-A pattern, and the remaining had the symmetrical variant pattern in both legs. Asymmetrical branching pattern was found in 7 cadavers composed of a common pattern in one leg and variant type in another leg (Table 2).

## Discussion

The popliteal artery branching patterns were classified by the classification of Kim et al. and Abou-Foul et al. into 12 subtypes. Type I-A is common in this study (90.7%). The major variant type in this study is type III-A, which is hypoplasia or aplasia of PT. This variant is found in 10 out of 162 limbs (6.2%). Type III-A pattern is also the most common variant pattern in South Korea, 5.1%^13^ and 4.8%^12^; Turkey, 3.5%^15^ and 5.6%^16^ and USA 3.8%^6^ (Table 3).

**Table 3:** Percentage of popliteal artery branching in published data.

Authors	Countries	Identification method	N (limbs)	Type I (%)	Type III-A (%)	Type III-B (%)	Type III-C (%)	Type IV (%)	Non-type III & IV (%)
Kim et al. 1989^6^	USA	Angiographic study	605	95.4	3.8	1.6	0.2	0	94.4
Manaster et al. 1990^17^	USA	Angiographic study	35	91.4	0	0	5.7	0	94.3
Young et al. 1994^3^	USA	Angiographic study	56	82.1	0	0	8.9	8.9	82.2
Blackwell et al. 1998^8^	USA	Angiographic study	38	81.6	0	2.6	2.6	5.3	89.5
Lutz et al. 1999^11^	Taiwan	Angiographic study	231	94.8	0.4	1.3	0.9	0.4	97
Seres et al. 2001^18^	Hungary	Angiographic study	64	84.4	3.1	1.6	1.6	1.6	92.1
Chow et al..2005^9^	USA	Angiographic study	32	81.3	0	9.4	3.1	3.1	84.4
Day et al. 2006^19^	United Kingdom	Angiographic study	1037	94.2	0.8	0.1	0.1	0	99
Lohan et al. 2008^20^	USA	Angiographic study	58	82.7	3.4	6.9	0	0	89.7
Kil et al. 2009^13^	South Korea	Angiographic study	1242	90.8	5.1	1.7	0.8	0	92.4
Sandhu et al. 2010^21^	USA	Angiographic study	53	83	7.5	1.9	0	0	90.6
Mavili et al. 2011^22^	Turkey	Angiographic study	535	88.2	3.7	2.2	0.2	0	93.9
Muhammad et al. 2012^23^	Saudi Arabia	Angiographic study	120	98.3	0.8	0.8	0	0	98.4
Akashi et al. 2013^24^	Japan	Angiographic study	38	100	0	0	0	0	100
Yanik et al. 2015^25^	Turkey	Angiographic study	116	88.8	3.4	0	0	0	96.6
Calisir et al. 2015^26^	Turkey	Angiographic study	636	91.4	2.7	0.9	0	0	96.4
Demirtaş et al. 2016^15^	Turkey	Angiographic study	1261	91.8	3.5	1.2	0.1	0.1	95.1
Celtikci et al. 2017^16^	Turkey	Angiographic study	863	84.5	5.6	5.1	1.5	0	87.8
Oner et al. 2020^27^	Turkey	Angiographic study	340	94.1	2	1.5	0.6	0	95.9
Piral et al. 1996^28^	France	Dissection	40	100	0	0	0	0	100
Choi et al. 2001^12^	South Korea	Dissection	63	95.2	4.8	0	0	0	95.2
Ozgur et al. 2009^29^	Turkey	Dissection	40	95	0	0	0	0	100
Holze et al. 2011^10^	Germany	Dissection	128	92.2	2.3	1.6	0	0	96.1
Muhammad et al. 2012^23^	Saudi Arabia	Dissection	40	97.5	2.5	0	0	0	97.5
Lappas et al. 2012^30^	Greece	Dissection	200	90	5	1.5	0	0	93.5
Olewnik et al. 2019^31^	Poland	Dissection	100	92	8	0	0	0	92
Ongsiriporn et al. 2021^a^	Thailand	Dissection	162	90.8	6.2	0	0	0.6	93.2

Each study was classified by adopting Kim et al. and Abou-Foul et al.’s classification. This current study mainly focused on type III and its subtypes and type IV, which are significant for operative planning, preventing severe donor site morbidities, and unusable fibula flap during fibular free flap harvesting.

^a^This current study.

The prevalence of type III-A in this study is higher than that found in the previous studies (Table 3). Thus, it suggests that the Thai population might have a higher prevalence of type III-A. As AT will be the only major blood supply for pedal circulation after FFF harvesting in type III-A pattern, donor site morbidity might occurs^10^. Hence, FFF harvest together with PR in type III-C variant, the dominant peroneal artery, and type IV, hypoplasia-aplasia of PR, may lead to distal limb ischemia of the donor’s leg or unusable fibular flap, respectively^11^.

As the AT arises as a branch of the PA while PT is formed by an anastomosis between PA and early distal femoral artery during embryogenesis^7^, it is hypothesized that the high prevalence of type III-A among studies might occur from abnormal formation of the arterial system during embryogenesis which results in higher prevalence of III-A than III-B in most studies (Table 3). Other variants found in this study are type I-B, II-B, and IV-A with the percentage of 1.2%, 1.2%, and 0.6%, respectively. Other patterns, which were not mentioned above, were not found in this study.

It was found that only 70 out of 81 cadavers have symmetrical common PA branching patterns compared to both legs due to the high number of this typical symmetrical pattern. FFF harvesting can be performed on at least one leg in most cases. However, the other 7, out of 81 cadavers, have asymmetrical patterns, common pattern in one leg and a variant in the other (Table 2). Therefore, if we found the variant pattern in one leg intraoperatively, the likelihood of the common pattern in the contralateral leg was 63.6%.

Typically, the distal AT and PT pulse are palpable at just above the foot's dorsum and medial malleolus, respectively^10^. Type III-A variant, hypoplasia or aplasia of the PT, leading to the replacement of distal PT by the PR ^6^. Our findings were also similar to prior study^6^. In this case, the pulse behind the medial malleolus might be palpable. For this reason, palpation of a distal pulse was unreliable for indicating the presence of PT.

With regard to other reports, the frequent variant was type III, as reported in South Korea, Turkey, and the USA. This suggested that the risk of serious complications after harvesting of FFF was similar among these countries (Table 3). In addition, type IV-A, hypoplastic PR, was found at a rate of 0.6% in our study and 0.1% in Turkey^15^.

Comparison between angiographic studies and cadaveric studies is shown in (Table 3). Type III-B and III-C patterns are mainly found in angiographic studies. The prevalence of these variants is different with statistically significant between angiographic study and cadaveric study (independent t-test at α = 0.05; p = 0.015 for type III-B and p = 0.009 for type III-C) (Table 4). We propose that atherosclerotic change of vessels might interfere with angiographic study’s interpretation, whereas cadaveric study dissects vessels from extraluminal. From this point and the fact that the peroneal artery is less involved by atherosclerotic change^5^, the prevalence of variant type III-B and III-C in the angiographic study might be acquired atherosclerosis of AT and PT. Hence, the decision-making for fibula free flap reconstruction in terms of the prevalence of popliteal artery branching variants should be considered by atherosclerosis risk. From the fact that the young patients who generally have less atherosclerosis risk might have benefited from cadaveric-based prevalence since the cadaveric studies investigate vessels from extraluminal which represent the congenital branching patterns of individuals. On the contrary, the old patients who mostly have atherosclerosis should be considered by the angiographic study because the acquired variants might play a role.

**Table 4:** Percentage of type III subtype variants between dissection and angiographic studies.

Identification method	Variant types		
	III-A (%)	III-B (%)	III-C (%)
Dissection (n = 40)	87.5	12.5	0
Angiographic study (n = 406)	58.3	31.0	10.6

The comparison data of type III and its subtypes from (Table 3), the percentage of each variant is compared between dissection and angiographic studies in type III-B and type III-C. The mean percentage of type III-B and type III-C in cadaveric dissection studies compared to angiographic studies are statistically significantly different. To illustrate, the mean percentage of type III-B are 12.5% and 31.0% in the cadaveric study and angiographic study, respectively (independent t-test, p = 0.015). Type III-C is in the same manner with 0% and 10.6% in the cadaveric study and angiographic studies, respectively (independent t-test, p = 0.009)).

**Figure 2:**
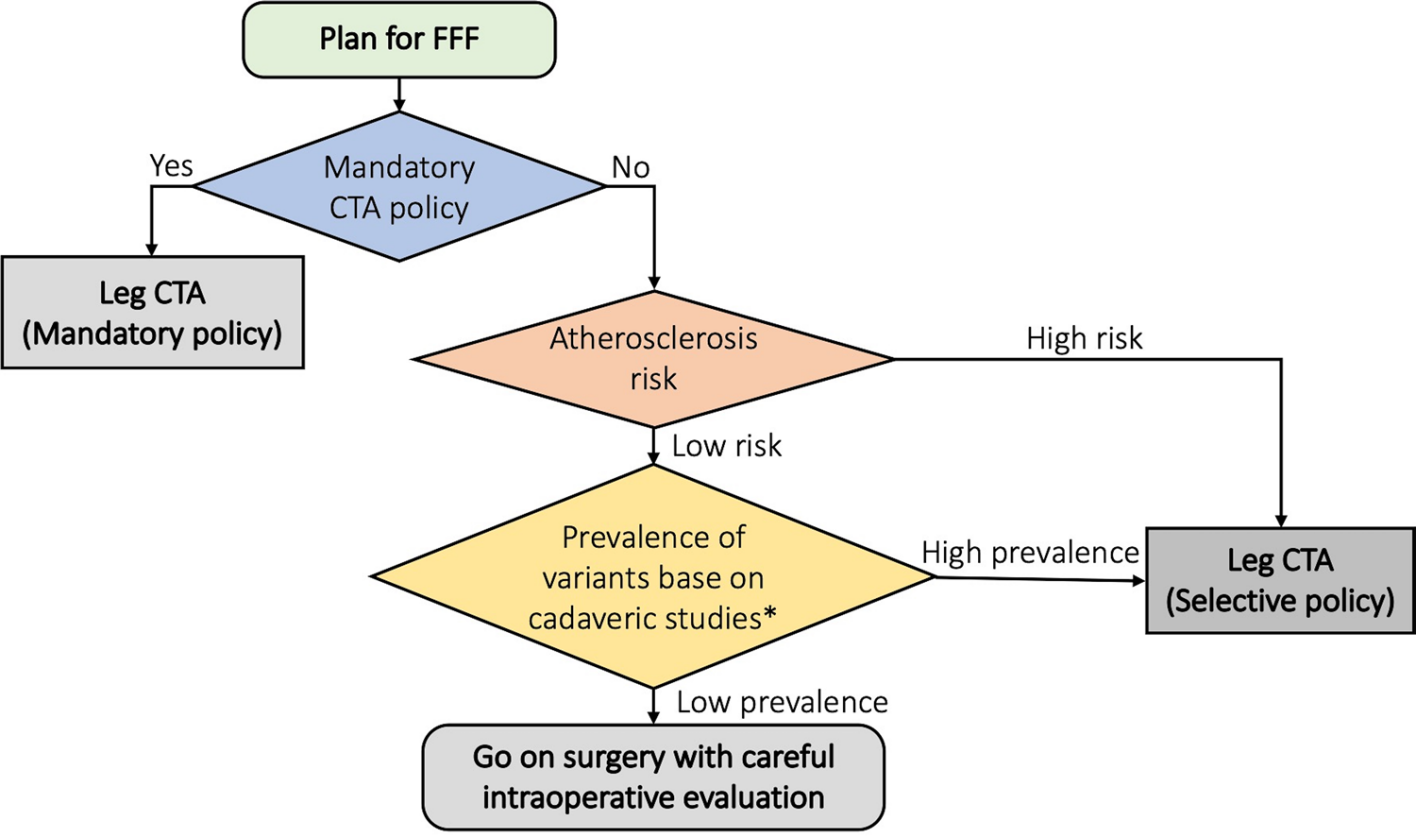
A schematic overview of pre-operative leg CTA in fibular free flap harvesting. If mandatory leg CTA policy is not applicable, which might be from the low prevalence of variants, limited contrast media amount from renal impairment, the leg CTA should be done following an atherosclerosis risk assessment. *The decision-making at this point should be based upon cost-effectiveness^8,14^ of leg CTA and prevalence in each population. To elaborate, in the low-risk for atherosclerosis group, the prevalence of variants should be referred from cadaveric studies since the prevalence is similar to the congenital prevalence. By contrast, patients at risk for atherosclerosis have a higher rate of acquired variants. *FFF* fibular free flap, *CTA* computed tomography angiography.

For the surgical planning of FFF harvesting, pre-operative leg CTA is mandatory in some centers. If mandatory leg CTA policy is not applicable, which might be from the cost-effectiveness^8,14^ in low prevalence of variants or limited amount of contrast media from patient’s underlying disease, the patient should be assessed by the atherosclerosis risk. For the patient who is at risk for atherosclerosis, he/ she should undergo pre-operative CTA due to the increased chance of acquired variants from atherosclerosis. However, the patient with low atherosclerosis risk and low prevalence of variants in the population can go on surgery with careful intraoperative evaluation. The prevalence of variants should be referred from cadaveric studies since the prevalence is similar to the congenital prevalence (Fig. 2).

However, further study of each branching type prevalence, which is categorized by age group, might help study the effects of atherosclerosis in PA and its collaterals. Then we might find a cutting point that can designate the benefit of using angiographic studies.

## Limitations of the study

There are some limitations to this study. First, this is an observational study using cadaveric dissection. The pre-mortem angiographic study of type III in patients could not be found in the first place. Thus, this was in contrast to what was found as type I in cadaveric dissection. It focused on demonstrating a case of acquired type III due to atherosclerosis change. Second, the number of cadavers in this study was relatively low compared to the number of cases in the angiographic study. Finally, this study highlighted popliteal artery branching patterns and arterial supply of fibula. Further study on the venous system of these areas should be considered.

## Materials and methods

This study was designed to meet criteria established in Helsinki declaration guidelines for research involving human subjects. Informed consent for donation to educational purposes and scientific research had been signed before death by the donors or after death by relatives, according to the local guidelines. This study was approved by the Institutional Review Board (IRB) of Faculty of Medicine Siriraj Hospital, Mahidol University (477/2564). The cadaver anonymity has been preserved. All methods were performed in accordance with the relevant guidelines and regulations described in the proposal submitted to the Ethical Committee.

The arteries of 168 lower limbs from 84 cadavers were dissected after a medical student’s routine cadaveric study, which has not been dissected in the interested area 162 lower limbs from 81 cadavers were recruited after exclusion criteria were applied. The cadavers in this study were embalmed immediately after death by injecting the solution containing formalin, glycerin, 95% ethanol, phenol, and water via the right femoral artery then immersed in the embalming solution consist of glycerin, carbolic acid, and water for 6 months.

Regarding the dissection methods, 162 lower limbs were separated from the trunk. Dissection of arteries and their collaterals was operationalized under Siriraj hospital’s laboratory manual. Veins in study area were sacrificed. The pattern of arterial branching and their relation to popliteus muscle was recorded.

Demographic data of cadavers and types of popliteal artery branching pattern according to classification by Kim et al.^6^ and Abou-Foul et al.^5^ were presented with descriptive statistics, including mean, range, and percentage. The differences in angiographic and cadaveric study variants were done using the Chi-square test and p-value under IBM SPSS Statistics for Windows, version 26.0 (IBM Corp., Armonk, NY, USA). A p-value lower than 0.05 was statistically significant.

## Conclusion

In order to harvest FFF safely, it is crucial to understand the arterial supply and anatomical variation of popliteal artery. Although type I-A is the most common pattern according to previous studies, it should be aware that there is a discrepancy in the prevalence of type III-B and III-C, which are mainly found in angiographic studies compared to cadaveric studies with statistically significant (p = 0.015 for type III-B and p = 0.009 for type III-C). Atherosclerosis might be the underlying of these differences.

The decision-making for pre-operative CTA for fibula free flap reconstruction in terms of the prevalence of popliteal artery branching variants should be considered by atherosclerosis risk. If pre-operative CTA policy is not mandatory, the authors suggest a selective CTA policy in patients at risk for atherosclerosis or high prevalence of variants in the population to select the suitable donor limb.
